# Clinical Evaluation of Nasopharyngeal, Oropharyngeal, Nasal Swabs, and Saliva for the Detection of SARS-CoV-2 by Direct RT-PCR

**DOI:** 10.3390/diagnostics12051091

**Published:** 2022-04-27

**Authors:** Sergei A. Kiryanov, Tatiana A. Levina, Vladislava V. Kadochnikova, Maria V. Konopleva, Anatoly P. Suslov, Dmitry Yu. Trofimov

**Affiliations:** 1 Federal State Budget Institution “National Research Centre for Epidemiology and Microbiology Named after Honorary Academician N.F. Gamaleya” of the Ministry of Health of the Russian Federation, 123098 Moscow, Russia; levina@dna-technology.ru (T.A.L.); m.konopleva@gamaleya.org (M.V.K.); suslov.anatoly@gamaleya.org (A.P.S.); 2 LLC “DNA-Technology”, 117587 Moscow, Russia; kadochnikova@dna-technology.ru (V.V.K.); dtrofimov@dna-technology.ru (D.Y.T.)

**Keywords:** COVID-19, SARS-CoV-2, direct RT-PCR, nasopharyngeal swab (NPS), oropharyngeal swab (OPS), nasal swab (NS), saliva sample (SS), sensitivity, specificity

## Abstract

Nasopharyngeal swab (NPS) and oropharyngeal swab (OPS) are the most widely used upper respiratory tract specimens for diagnosis of SARS-CoV-2 using RT-qPCR. In contrast, nasal swab (NS) and saliva (SS), recently recommended by the WHO, are rarely used, and their test accuracy is limited. The method for direct RT-PCR detection of SARS-CoV-2 does not require an RNA extraction and is faster and easier than standard RT-PCR tests with RNA extraction. This study aimed to compare the diagnostic performance of upper respiratory tract samples for SARS-CoV-2 detection using the direct RT-PCR without preliminary heat inactivation. Here we report the application and validation of direct RT-PCR SARS-CoV-2 RNA on 165 clinical specimens of NPS/OP, and 36 samples of NS/NPS and 37 saliva samples (for the latter with prior deproteinization). The overall sensitivity estimates were 95.9%, 94.2%, 88.9%, and 94.6% for NPS/OPS/NS/SS samples, respectively, and the specificity was 100% against standard RT-PCR with RNA extraction. Overall, NS and SS testing by direct RT-PCR had sufficient sensitivity to detect SARS-CoV-2. They can be acceptable alternative to NPS/OPS for rapid detection of SARS-CoV-2 infections in future.

## 1. Introduction

The pandemic of coronavirus disease 2019 (COVID-19) caused by severe acute respiratory syndrome coronavirus 2 (SARS-CoV-2) still puts a tremendous strain on public health worldwide. The availability of accurate COVID-19 diagnostic tests is one of the critical steps of tackling the pandemic [1].

Real-time reverse transcription-quantitative polymerase chain reaction (RT-qPCR) is the current gold standard for the diagnosis of COVID-19 from NPS and/or OPS specimens, collected in viral transport medium, and is included in the protocols recommended by the WHO and other international organizations [2,3].

The commonly used protocols require a ribonucleic acid (RNA) extraction step, which may be seen as a critical aspect in the massive laboratory testing process, especially in the SARS-CoV-2 era [4]. The huge demand for RT-PCR testing of SARS-CoV-2 has led to a worldwide shortage of laboratory reagents, including RNA isolation kits, which remains a major problem. To overcome the shortage of reagents and expand the capacity for SARS-CoV-2 testing, approaches skipping the RNA extraction step have been developed [5,6,7].

Several groups have shown the feasibility of extraction-free RT-PCR for SARS-CoV-2 mainly on NPS with a preliminary heat inactivation step at 95 °C for 5 min or 70–75 °C for 10–15 min [7,8,9,10]. It has also been suggested that the absence of the preliminary heat inactivation step in direct RT-PCR may lead to insufficient release of RNA from the virion and a decrease in the efficiency of RNA detection by direct RT-PCR [10]. On the other hand, the heat treatment step (95 °C for 5 min), a prior direct RT-PCR aimed to lyse epithelial cells, may, however, reduce the detection rate of the samples with low viral load due to the inverse effect of thermolysis on RNA integrity as was noted by others [11,12,13].

For initial diagnostic testing for current SARS-CoV-2 infections, CDC recommended collecting and testing upper respiratory tract specimens such as NPS and OPS as well mid-turbinate NS and saliva [14]. The FDA also states that that “more data are necessary to better understand the performance when using specific saliva collection or other specimen types for COVID-19 testing” [15]. Moreover, self-collected nasal swabs and saliva are less invasive and easy to collect than NPS/OPS and thus more suitable for mass screening than NPS sampling [16,17].

OPS and NPS have been a preferred specimen type in Russia for SARS-CoV-2 molecular diagnosis since the beginning of the pandemic. However, the invasiveness and painful of this sampling are of its disadvantages. Additionally, variable collection quality and demand for supervised collection by trained personnel create obstacles for the scalability of this testing [18,19]. Recently recommended non-invasive sampling such as saliva [20,21] or nasal swabs [22] combined with rapid direct COVID-19 RT-PCR assays [23] could potentially enable broader SARS-CoV-2 testing of at-risk populations. Standard RT-PCR assays with RNA extraction using NS and SS have been rarely applied in Russia, data about the diagnostic sensitivity for direct RT-PCR is still limited.

The aim of this study was to evaluate the diagnostic performance of the direct multiplex RT-PCR method, without RNA extraction, on clinical specimens of upper respiratory tract: NPS, OPS, NS, and saliva, and to compare with the performance of the traditional method based on RNA extraction, in a real-world setting.

## 2. Materials and Methods

### 2.1. Upper Respiratory Clinical Sample Collection and Preparation

Upper respiratory tract specimens, including nasopharyngeal, oropharyngeal, and nasal swabs as well as saliva were collected and were a part of routine diagnostics to detect SARS-CoV-2. Informed voluntary consent was obtained from all involved patients. The study was carried out according to the guidelines of the World Medical Association’s Declaration of Helsinki.

We were able to collect 122 OPS/NPS, 36 NS/NPS, and 37 saliva/OPS paired SARS-CoV-2 positive samples, and 43 SARS-CoV-2 negative samples. Symptom status of patients was not recorded at the time of the study swab. OPS collection involved swabbing of the palatine arches and the back wall of the oropharynx. The NPS sampling was performed through one nostril. For NS collection, one nostril was swabbed to a depth of at least 2 cm and rotated 3 times. Saliva specimens were diluted with 3-fold low saline buffer for an endpoint volume of 1.0 mL to reduce the viscosity of the sample. Saliva suspension was centrifuged at 2000× *g* for 5 min at 4 °C, and the supernatant was treated with Proteinase K at 55 °C for 15 min following heat inactivation at 95 °C for 5 min. Finally, 10 µL of saliva suspension was used in direct RT-PCR. One oropharyngeal and one nasopharyngeal swab from each patient or one nasopharynx and one nasal swab from each patient or oropharyngeal and saliva swab were placed into 0.5 mL of STORE-F transport medium (DNA-technologies, Moscow, Russia) to obtain a sample to set either direct (without RNA extraction) or standard RT-PCR. All types of samples were stored at +4 °C for a maximum of 5 days until processing. The mean number of days from diagnostic swab to study swabs was 3.1 (range 1–5).

### 2.2. Viral RNA Extraction

All specimens were collected from patients in Moscow and the Moscow region, Russia. Samples were centrifuged at high speed (>2000× *g*) for 5 min to pellet cellular debris and then stored at +4 °C until qPCR was performed. RNA extraction was performed using manual extraction protocol with a triazole based PREP-NA kit (DNA Technologies, Moscow, Russia) according to the manufacturer’s manual. Viral RNA extract was eluted in 100 μL of the elution buffer, and an RT-PCR assay was performed.

### 2.3. SARS-CoV-2 RNA Detection Using Standard RT-PCR

In addition, 10 μL of the RNA elute were mixed with 40 μL of PCR master mix SARS-CoV-2 Lite Real-Time PCR Detection Kit (DNA-Technologies, Moscow, Russia) to make a total of 50 μL of reaction volume. The master mix contained components as following PCR buffer with magnesium salt, primers, and probes. Ferment mix included reverse transcriptase, RNAsin, and DNA polymerase. RT-PCR applying SARS-CoV-2 Lite Real-Time PCR Detection Kit was able to target the SARS-CoV-2 *E* and *RdRp* genes with detection in the FAM channel. The analytical sensitivity (LOD) reported was 10 copies/reaction. Automatic analysis was performed on DTprime Real-Time Thermal Cycler using the DTmaster software for the DNA Technologies kit, included in the DT Lite system (DNA Technologies, Moscow, Russia). The result was interpreted as a positive if threshold value (Ct value) <40 for all two target genes. The PCR conditions consisted of 1 cycle of 5 min at 60 °C, 2 min at 94 °C and followed by 45 cycles of 5 s at 94 °C, 10 s at 58 °C and 10 s at 64 °C.

### 2.4. Detection of Viral RNA by Direct RT-PCR Method without RNA Extraction

Direct RT-qPCR without RNA extraction was performed using the same SARS-CoV-2 Lite Real-Time PCR Detection Kit (DNA Technologies, Moscow, Russia) according to the manufacturers’ instructions. Before analysis, the swabs were spin centrifugated at >2000× *g* for 5 min and 10 µL were used in RT-PCR without prior heat inactivation. In addition, 10 μL of the sample in transport medium STORE-F were mixed with 40 μL of PCR master mix SARS-CoV-2 Lite Real-Time PCR Detection Kit to make a total of 50 μL of reaction volume. Direct RT-PCR applying SARS-CoV-2 Lite Real-Time PCR Detection Kit was conducted with the same protocol as the standard RT-PCR. A positive result, indicating the presence of SARS-CoV-2 RNA, was determined according to the Ct value. Ct values of <40 with the target primer/probe set for SARS-CoV-2 indicated virus presence. Positive (SARS-CoV-2 Lite RNA Positive Control) and negative controls (nuclease-free water) were used to control and exclude possible false-negative results. MS2 phage served as an internal control for control reverse transcription and the amplification of material collected in each sample.

### 2.5. Statistical Analysis

The correlation between the Ct values for both direct and standard methods was calculated by Pearson’s rank correlation using the Excel Analysis ToolPak. *p*-values of <0.05 were considered statistically significant. The sensitivity and specificity values and corresponding confidence intervals were calculated with assistance of an online tool (Available online: https://www.medcalc.org/calc/diagnostic_test.php (accessed on 18 March 2022)).

## 3. Results

### 3.1. Sensitivity Assessment of Direct RT-PCR on Clinical Oropharyngeal and Nasopharyngeal Swabs

A total of 165 tested samples were stored in STORE-F at +4 °C before PCR as recommended by the manufacturer. To evaluate the ability of direct RT-PCR to detect SARS-CoV-2 RNA without RNA isolation, 122 clinical OPS/NPS pair samples from patients with SARS-CoV-2 and 43 SARS-CoV-2 negative ones were performed. The standard RT-PCR protocol served as a reference to evaluate the performance of the direct PCR protocol. Threshold cycle values ranging from 18.3 to 40.5 were previously obtained by standard RT-qPCR using SARS-CoV-2/SARS-CoV Multiplex Real-Time PCR Detection Kit (DNA Technologies, Moscow, Russia) with RNA isolation from a 200 µL sample in transport medium and concentrating in 50 µL of the TE, and subdivided into samples with high viral load (Ct < 24), medium (Ct 25–34), and low (Ct > 35). For direct RT-PCR, 10 µL of swab specimen stored in STORE-F transport medium were used. In parallel, a 100 µL aliquot of each sample was used for RNA isolation without subsequent concentration and the equivalent of 10 µL of the original swab diluent used for RT-PCR, allowing a one-to-one comparison with direct RT-PCR. The overall diagnostic sensitivity of direct RT-PCR on OPS and NPS samples relative to the standard with RNA isolation was 94.2% (95% CI: 86.1–97.6%) and 95.9% (95% CI: 91.2–98.4%) (Table 1). Ct (mean ± standard deviation) values for both targets *E* (envelope) and *RdRP* (RNA-dependent RNA polymerase) genes by OPS (27.2  ±  5.1) and NPS (26.8  ±  4.9) testing were similar. Differences in mean Ct values for the target genes in direct RT-PCR on both OPS and NPS were not statistically significant (*p*  =  0.59). The direct RT-PCR protocol using OPS showed 95% concordance with the standard protocol and mean difference in Ct values of 1.69 for both *E* gene and the *RdRp* gene targets. The direct RT-PCR protocol using NPS showed 98% concordance with the standard protocol and mean difference in Ct values of 1.62 for both *E* gene and the *RdRp* gene targets.

Of the 122 positive samples, 6 and 5 were false negatives by the direct RT-PCR on OPS and NPS samples, respectively, compared to the standard protocol with RNA extraction. All but one OPS and NPS samples not detected by direct RT-PCR corresponded to Ct = 39.0 and Ct = 40.5 in standard RT-PCR with RNA isolation, i.e., were treated as samples with an extremely low viral load. Only one OPS sample appears to be a false negative with a corresponding Ct value of 33 in standard RT-PCR. Triple dilution and deproteinization with Proteinase K before repeated direct RT-PCR allowed the detection of SARS-CoV-2 in this sample (Ct = 34.4). The presence of saliva inhibitory proteins and salts in this OPS sample assumed to cause a failure to detect SARS-CoV-2 RNA in the first setting.

### 3.2. Sensitivity Assessment of Direct RT-PCR on Clinical Nasal and Nasopharyngeal Swabs

Next, we tested NS and NPS samples in parallel followed by direct RT-qPCR versus standard RT-qPCR with RNA extraction on a collection of 68 patients. Of these subjects, 36 (52.9%) were found to be positive and 32 (47.1%) negative for SARS-CoV-2 RNA using the standard RT-qPCR with RNA extraction from NPS. The sensitivity of SARS-CoV-2 RNA detection by direct RT-PCR from 36 nasal samples was 88.9% (95% CI: 81.3–94.1%), and specificity was 100.0% (95% CI: 94.6–100.0%) (Table 2). Thus, we found that the % positive NS (88.9% (95% CI: 76.3–93.2%)) was lower than % positive NPS swab (94.4% (95% CI: 86.1–97.8%)). Upon comparison of direct RT-qPCR with standard RT-qPCR, NS showed an agreement of 94.1% for both positive and negative samples. The direct RT-PCR protocol using NPS showed 97% concordance with the standard protocol. Upon comparison of direct RT-qPCR using NS versus NPS, an agreement was 97.0%. Ct (mean ± standard deviation) values for both targets *E* and *RdRP* genes by NPS (26.9  ±  4.9) and NS (27.6  ±  5.2) were similar. Differences in Ct values for the target genes were not statistically significant (*p*  =  0.59).

Of the 36 positive results from direct RT-PCR protocol on NS, 9 samples (25.0%) exhibited low viral loads (Ct > 35). Only 4 and 2 NS and NPS samples failed to be detected by direct RT-PCR. However, one NS sample was false negative by direct RT-PCR, while it has been detected in paired NPS by direct RT-PCR (Ct = 32.6) and by standard RT-PCR (Ct = 31). This result probably indicated either the uneven virus distribution in the NS/NPS or inhibitory effect of mucus in the NS sample.

**Figure 1:**
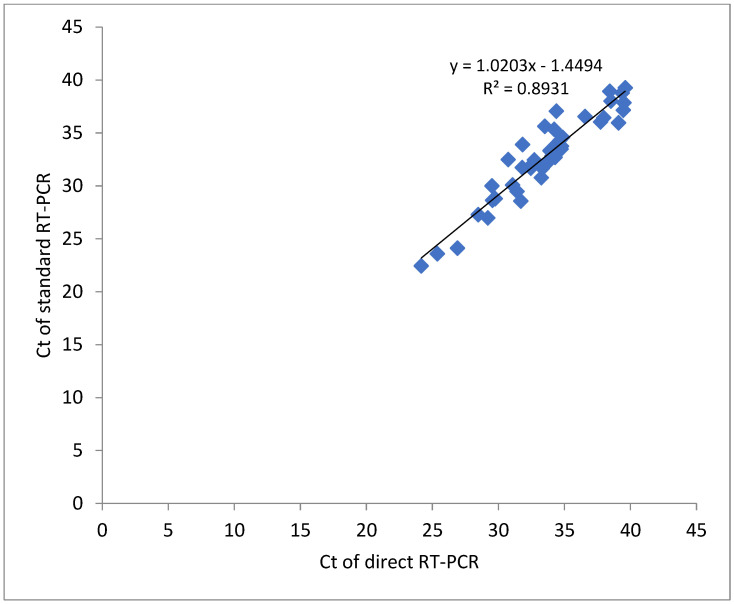
Correlation of Ct values detected for NS samples in direct and standard SARS-CoV-2 RT-qPCR methods.

Nasal swab sampling with direct RT-PCR correlated with the standard RT-PCR across the whole range of Ct values as shown in Figure 1. The correlation coefficient (R^2^ = 0.893) may be associated with the analysis of normalized Ct values.

### 3.3. Direct RT-PCR on Saliva Samples

Next, the effectiveness of direct multiplex RT-PCR using saliva samples was tested. Previously, we have found that, to reduce the inhibitory effect of salivary proteins before performing direct RT-PCR on saliva samples, it is necessary to add Proteinase K (+ProK) to the samples and deproteinize for 15 min at 55 °C, followed by proteinase inactivation at 95 °C for 5 min. Using only heat treatment (95 °C for 5–10 min) and fold dilution of the original saliva sample resulted in a significant number of samples non-detectable or invalid due to the lack of signal for both target targets and internal control. In addition to deproteinization, the second factor improving the efficiency of direct RT-PCR was at least 3-fold dilution of the original saliva sample to reduce viscosity as recommended [24].

We compared the effectiveness of direct RT-PCR on saliva samples with Proteinase K treatment (Saliva/ProK) and with Proteinase K and 3-fold dilution (saliva/ProK+:3) against standard RT-PCR on 37 positive and 24 negative saliva samples. The sensitivity of SARS-CoV-2 RNA detection by direct RT-PCR on both variants was 91.9% (95% CI: 84.1–95.2%) and 94.6 (95% CI: 86.2–97.9%), respectively. Specificity was 100.0% (95% CI: 94.6–100.0%) (Table 3). A few false-negative saliva samples in direct RT-PCR corresponded to samples with a Ct value ranging of 39.2 to 40.0 in standard RT-PCR, indicating a viral load close to the LOD level (10 copies/reaction). Upon comparing direct RT-qPCR with standard RT-qPCR, SS with dilution showed an agreement of 97.0%, while SS without dilution –95.0%. Ct (mean ± standard deviation) values for both targets *E* and *RdRP* genes for SS without dilution (27.3  ±  4.8) and SS with dilution (28.1  ±  5.2) were similar. Thus, we found that the % positive saliva samples (94.6%) were similar to % positive OPS swab (94.2%).

**Figure 2:**
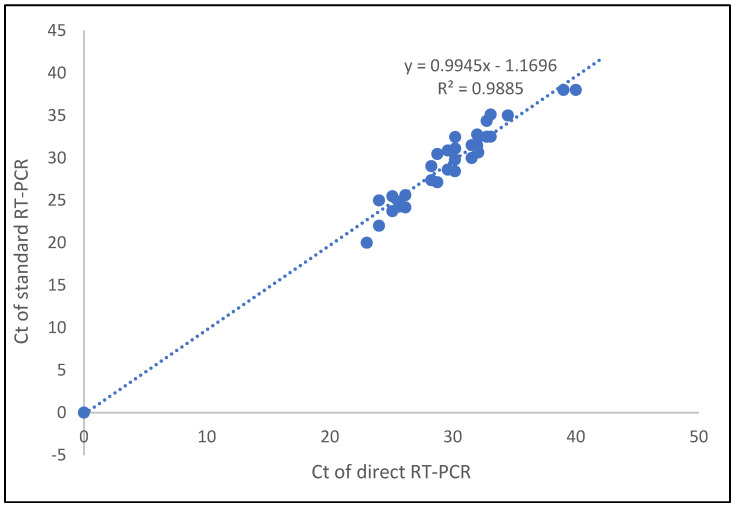
Correlation of Ct values detected for saliva samples in direct and standard SARS-CoV-2 RT-qPCR methods.

For the Ct value correlation between the direct method and the standard method for saliva samples, the correlation coefficient of the E gene and RdRP genes was 0.987. Thus, Ct values of the detected genes were highly correlated between the two methods (Figure 2).

## 4. Discussion

In this study, we demonstrated that the direct RT-PCR method for detection of SARS-CoV-2 may be a valuable alternative to a standard RT-PCR method with RNA extraction. Our data describe quite sensitive direct detection of SARS-CoV-2 RNA by multiplex RT-qPCR without heat shock compared to standard RT-PCR on real-world upper respiratory tract samples including NPS, OPS, nasal (mid-turbinate) swabs, and saliva. Regarding COVID-19, all of these upper respiratory tract samples are recommended for SARS-CoV-2 RT-qPCR detection [2].

All four sample types tested by the direct RT-PCR appeared to capture slightly lower % positives: NPS (95.9% (95% CI: 91.2–98.4%)) and OPS (94.2% (95% CI: 86.1–97.6%)), nasal swabs (88.9% (95% CI 76.3–93.2%)), and saliva (94.6 (95% CI: 86.2–97.9%)) in comparison to standard RT-PCR with RNA extraction. We found that the detection rate for NPS seemed to be slightly higher compared to OPS, but it was estimated on a small number of positive patients.

Previous studies have reported opposing conclusions regarding whether nasal sampling is concordant or discordant with NPS [25,26]. Here, we report that the molecular detection of SARS-CoV-2 by direct RT-PCR on using NS was slightly lower for the detection using NPS. We observed that the discordant samples in pair NPS+/NS- had low viral load on the NPS. This may well be uneven distribution of the virus through the upper respiratory tracts (nasal and nasopharyngeal) or an inhibitory effect of mucosal secretion in the NS sample. Our results strongly suggest that the difference in NS and NPS/OPS performance depends on the viral load in positive samples, with high concordance for medium-to-high viral loads (Ct < 35) and low concordance for very low viral loads, which may lie close to the LOD of the direct RT-PCR assay. On the other hand, sequential sampling (NPS first, then NS or NPS first, then OPS or OPS first, then saliva) may have also affected performance with subsequent swabs. Overall, SARS-CoV-2 detection using direct RT-PCR from nasal samples showed appropriate sensitivity and high specificity.

Using direct RT-PCR with saliva specimens, we found similar performance compared to NPSs and very similar to OPS (94.6 (95% CI: 86.2–97.9%)) versus 95.9% (95% CI: 91.2–98.4%) and 94.2% (95% CI: 86.1–97.6%), respectively, and only the absence of sufficient dilution of saliva sample resulted in a noticeably lower rate of detection (91.9% (95% CI, 84.1–95.2%)). Several previous studies reported that saliva specimens show similar sensitivity to NPS and OPS and support our data [16,24,27,28,29].

Our results provide data that the direct detection of SARS-CoV-2 without heat pretreatment might be an acceptable alternative to the standard RT-PCR with RNA extraction for SARS-CoV-2 detection, although very low viral loads samples would not be detected. Several direct RT-PCR protocols have been described and validated using a pre-heat step at high temperatures above 95 °C for direct RT-qPCR without RNA extraction [8,9,10,30]. The heat pretreatment procedure introduced in order to lyse epithelial cells may, however, reduce sensitivity and increase false negative rate as it was noted for temperature regimen 95 °C [12,13,31] as well as 70–75 °C [9]. In our study, the direct RT-qPCR was in complete agreement with the standard RT-PCR method with all four types of upper respiratory tract samples. However, the detection accuracy with NS samples highly depends on the viral load in the sample, and several false negative results are possible in the samples with very low viral load close to the limit of detection.

This study has several limitations, especially regarding the sample collection conditions for SARS-CoV-2 RT-qPCR. We did not have any information regarding whether the participants were symptomatic at the time of swabbing, which could impact viral shedding dynamics for different types of samples. The collectors did not provide sufficient information about the collection day of infection and other key issues (asymptomatic or symptomatic, early or late testing from symptom onset, severity or mild status). We also were not able to collect all samples in all four types for each patient. Secondly, we provided evidence about the sensitivity of NS and saliva samples on a limited number of patients with SARS-CoV-2 positive and negative diagnosis. We did not have enough participants to say with 95% certainty that NS was 8% less sensitive than NPS or that saliva was as sensitive as OPS. We cannot exclude that sensitivity values would be lower on aa larger group of participants. Another limitation to the saliva sampling is the fact that some patients failed to produce enough saliva. Food or drug contaminants in saliva might also deteriorate the quality of the assays. As for NS samples, it is not always possible to evaluate the influence of variable concomitant inhibitory factors to yield sensitivity of direct RT-PCR. Such inhibitory factor is excessive mucus secretion caused by rhinitis. An additional limitation of our method is safety concerns for healthcare professionals being potentially exposed to the SARS-CoV-2 virus shortly before PCR set up, although now direct RT-PCR is a routinely used method by trained personnel in the special facilities in Russia.

Several groups recently reported about sufficient sensitivity of standard and direct RT-PCR on self-collected NS and saliva samples in SARS-CoV-2 diagnosis among symptomatic patients [23,32]. Authors described the opportunity of self-collection sampling as a vivid alternative to routine collection by trained personnel. Due to current guidelines of the Russian Ministry of Health on diagnosis and treatment of novel coronavirus infection (COVID-19) [33] to make sample collection by health-care-workers only, we did not make an attempt to evaluate the performance of our direct RT-PCR on self-collected samples.

## 5. Conclusions

In conclusion, we have shown that a direct RT-qPCR method without RNA extraction can effectively detect SARS-CoV-2 RNA from upper respiratory tract specimens including nasal swabs and saliva. The direct RT-PCR method had sufficient sensitivity to detect SARS-CoV-2 with viral loads that correlate with the presence of an infectious virus. We propose that the use of non-invasive nasal swabs and saliva combined with direct RT-qPCR has a potential to provide the diagnostic utility of this approach for future SARS-CoV-2 testing.

## Figures and Tables

**Table 1:** Detection sensitivity of direct RT-qPCR versus standard RT-qPCR on oropharyngeal and nasopharyngeal swabs containing a range of SARS-CoV-2 viral RNA loads.

Relative SARS-CoV-2 RNA Load ^a^	Direct RT-PCR (10 μL/rxn) OPS ^b^	Direct RT-PCR (10 μL/rxn) NPS ^c^	Standard RT-PCR (10 μL/rxn) ^d^	Mean ΔCt Direct RT-PCR OPS ^e^	Mean ΔCt Direct RT-PCR NPS ^f^
High (Ct < 24)	24/24 (100%)	24/24 (100%)	24/24 (100%)	1.79	1.74
Medium (Ct 25–34)	82/83 (98%)	83/83 (100%)	83/83 (100%)	1.84	1.81
Low (Ct > 35)	9/15 (60%)	10/15 (67%)	14/15 (93%)	1.43	1.32
Total Sensitivity	115/122 (94.2%)	118/122 (95.9%)	121/122 (99.2%)	<1.69>	<1.62>
Specificity	43/43 (100%)	43/43 (100%)	43/43 (100%)		

^a^ Cts determined by standard RT-qPCR using the equivalent of 20 μL of RNA extracted from an NPS. The CoV-2 E and RdRp primer/probe set was used for the RT-qPCR reactions; ^b^ The volume of OPS diluent in STORE-F was loaded into a direct RT-qPCR master mix with the same SARS-CoV-2 E and RdRp primer/probes; ^c^ The volume of NPS diluent in STORE-F was loaded into direct RT-qPCR master mix with the same SARS-CoV-2 E and RdRp primer/probes; ^d^ RNA was extracted from 100 μL of OPS diluent and the equivalent of 10 μL OPS diluent was loaded into RT-qPCR; ^e^ Mean ΔCt for both *E* gene and the *RdRp* gene targets determined by direct RT-PCR using OPS samples vs. standard RT-qPCR; ^f^ Mean ΔCt for both *E* gene and the *RdRp* gene targets determined by direct RT-PCR using NPS samples vs. standard RT-qPCR.

**Table 2:** Detection sensitivity of direct RT-qPCR versus standard RT-qPCR on nasal swabs and nasopharyngeal swabs containing a range of SARS-CoV-2 viral RNA loads.

Relative SARS-CoV-2 RNA Load ^a^	Direct RT-PCR (10 μL/rxn) NS ^b^	Direct RT-PCR (10 μL/rxn) NPS ^c^	Standard RT-PCR (10 μL/rxn) ^d^
High (Ct < 24)	9/9 (100%)	9/9 (100%)	9/9 (100%)
Medium (Ct 25–34)	17/18 (94.4%)	18/18 (100%)	18/18 (100%)
Low (Ct > 35)	6/9 (66.7%)	7/9 (66.7%)	9/9 (100%)
Total Sensitivity	32/36 (88.9%)	34/36 (94.4%)	36/36 (100%)
Specificity	32/32 (100%)	32/32 (100%)	32/32 (100%)
Mean Ct ^e^	27.6 ± 5.2	26.7 ± 4.9	25.2 ± 5.4

^a^ Cts determined by standard RT-qPCR using the equivalent of 20 μL of RNA extracted from an NPS. The CoV-2 E and RdRp primer/probe set were used for the RT-qPCR reactions; ^b^ The volume of NS diluent in STORE-F was loaded into direct RT-qPCR master mix with the same SARS-CoV-2 E and RdRp primer/probes; ^c^ The volume of NP diluent in STORE-F was loaded into direct RT-qPCR master mix with the same SARS-CoV-2 E and RdRp primer/probes; ^d^ RNA was extracted from 100 μL of NPS diluent and the equivalent of 10 μL NPS diluent was loaded into RT-qPCR; ^e^ Mean Ct for both targets *E* gene and the *RdRp* gene determined by direct RT-PCR and standard RT-qPCR on NS and NPS specimens.

**Table 3:** Detection sensitivity of direct RT-qPCR versus standard RT-qPCR on saliva and 3-fold diluted saliva samples containing a range of SARS-CoV-2 viral RNA loads.

Relative SARS-CoV-2 RNA Load ^a^	Direct RT-PCR (10 μL/rxn) Saliva/ProK ^b^	Direct RT-PCR (10 μL/rxn) Saliva/ProK+:3 ^c^	Standard RT-PCR (10 μL/rxn) ^d^
High (Ct < 24)	9/9 (100%)	9/9 (100%)	9/9 (100%)
Medium (Ct 25–34)	19/19 (100%)	19/19 (100%)	19/19 (100%)
Low (Ct > 35)	6/9 (66.7%)	7/9 (77.8%)	9/9 (100%)
Total Sensitivity	34/37 (91.9%)	35/37 (94.6%)	37/37 (100%)
Specificity	24/24 (100%)	24/24 (100%)	24/24 (100%)
Mean Ct ^e^	27.3 ± 4.8	28.1 ± 5.2	26.0 ± 4.8

^a^ Cts determined by standard RT-qPCR using the equivalent of 20 μL of RNA extracted from an NPS. The SARS-CoV-2 E and RdRp primer/probe set was used for the RT-qPCR reactions; ^b^ The volume of saliva sample in STORE-F was loaded into direct RT-qPCR master mix with the same SARS-CoV-2 E and RdRp primer/probes; ^c^ The volume of saliva triple-diluent in STORE-F was loaded into direct RT-qPCR master mix with the same SARS-CoV-2 E and RdRp primer/probes; ^d^ RNA was extracted from 100 μL of NPS diluent and the equivalent of 10 μL NPS diluent was loaded into RT-qPCR featuring the same primer/probe set; ^e^ Mean Ct for both targets *E* gene and the *RdRp* gene determined by direct RT-PCR and standard RT-qPCR on saliva samples.
